# Electrical percolation threshold of carbon black in a polymer matrix and its application to antistatic fibre

**DOI:** 10.1038/s41598-019-42495-1

**Published:** 2019-04-19

**Authors:** Hyun-Jung Choi, Moo Sung Kim, Damiro Ahn, Sang Young Yeo, Sohee Lee

**Affiliations:** 1 Technical Textile R&D Group, Korea Institute of Industrial Technology, 143 Hanggaulro, Sangnok-gu, Ansan-si, Gyeonggi- do 15588 Republic of Korea; 2 Department of Clothing and Textiles, Research Institute of Natural Science, Gyeongsang National University, 501 Jinju-daero, Jinju-si, South Gyeongsang Province 52828 Republic of Korea

**Keywords:** Electronic properties and materials, Composites

## Abstract

In this study, using three types of resins (each with unique material properties) as a matrix, and carbon black (CB) as a conductive additive, conductive fibres were fabricated through a melt-spinning process. An examination of the electrical conductivity revealed that a CB/polyethylene terephthalate (PET) composite had a low percolation value of 0.58 wt%, and thus the highest conductivity of the three resin types. These results indicate that CB/PET fibres could be used to manufacture antistatic fabrics.

## Introduction

Consumer demand for smart equipment continues to increase, leading to research being carried out to devise fibres with functions that go beyond simple body protection and clothing design, as well as new functions that will be realized by combining such fibres with information and communications technology, as well as other developments. The range of application fields depends on the conductivity of the fibre, and it is even possible to apply conductive materials to electrostatic discharge products (1–10^11^ Ω/sq)^1,2^, electromagnetic disturbance products, (EMI: 1–10^−2^ Ω/sq)^3–5^, etc. Wearable materials include films^6–9^, fibres^10,11^, fabrics^12,13^, and other forms. To be practical, wearable devices must be flexible and electrically conductive. Fibres, being elastic, are the most appropriate type of material for this application. In particular, conductive fibres are seen as being a vital material for the fabrication of wearable components, yet relatively little research is being undertaken to investigate fibre-based electrodes and wires that can be used in practice. Most research conducted to date has produced conductive fibres by adding carbon black^2,14^, carbon nanotubes^2,15,16^, graphite^17–19^, or metal powder^20,21^, to polymers to produce conductive fibres.

Of the above-mentioned materials, carbon black (CB) is most commonly used as an agent for making plastic materials conductive. If carbon black is mixed into a polymer matrix, not only does it increase the mechanical strength, its high specific surface area means that, even in small amounts, it forms a conductive network which greatly increases the conductivity of the plastic^22^. Research into CB/polymer conducting composites is currently underway in a variety of fields. Many studies have examined the use of different types of polymer matrix with carbon black, in particular in terms of how the percolation threshold is affected^23–25^. Also, several researchers have examined the use of a small amount of mixed CB/carbon nanotube conducting filler to decrease the percolation threshold^26–28^. It has been reported that the percolation threshold of many composites corresponds to a conductive filler content of approximately 3–15 wt%^29^.

To apply such a polymer-composite fibre with conductive additive to a range of different fields, the fibre must first be formed. Solution spinning^30^, metal coating^31^, electrospinning^32^ and bubbfil spinning^33^ have all been used to fabricate conductive fibres. These processes all produce high-conductivity fibres, but none are suitable for mass production as the processes are complicated and costly to implement.

In the case of melt spinning, it is difficult to control the mixing ratio, screw speed, feed speed, and dispersion, so it has not proven popular for the fabrication of conductive fibres^34^. However, in comparison with fibres produced using other processes, those produced by melt-spinning exhibit excellent mechanical strength. Furthermore, given that large-scale production as well as a reduction in unit cost is possible, this method warrants further research.

Therefore, in the present study, three different polymer matrixes (polypropylene (PP), polyethylene terephthalate (PET), and polyamide (Nylon)) were combined with carbon black to produce a composite film using a melt-spinning process. The change in the percolation threshold according to the polymer’s properties was then determined. Also, a detailed study was made of the influence of the morphology and conductivity of the composite on the polymer matrix. Based on the results obtained for the percolation threshold, PET (which offers the best conductivity) was used to fabricate a CB/PET composite fibre. This fibre was woven into a fabric, the conductivity of the fabric was evaluated, and the possibility of applying it as an antistatic charge fabric was investigated.

## Results and Discussion

### Characteristics of CB/Polymer composites

**Figure 1:**
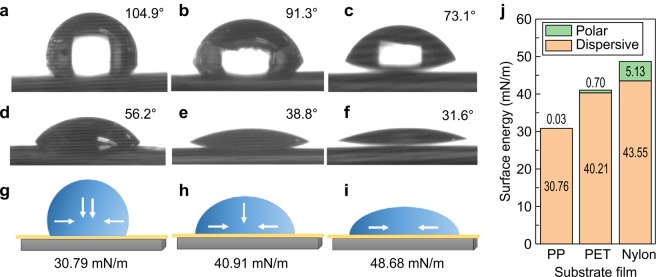
Polymer matrix surface energy. Contact angles of water droplet on (**a**) PP film, (**b**) PET film, (**c**) Nylon film and diiodomethane drop on (**d**) PP film, (**e**) PET film, and (**c**) Nylon film. Surface energy of (**g**) PP film, (**h**) PET film, and (**i**) Nylon film. (**j**) Polar and dispersive values of surface energy.

To calculate the polymer matrix surface energies of the three types of polymer matrix used in the experiment, that is, PP, PET, and Nylon, water and diiodomethane were used and the contact angle was measured as shown in Fig. 1. The tangent angle between the solution droplet and polymer substrate surface was measured as the contact angle. The contact angle was measured five times and the average value was used. The contact angles of the water droplets increased in the following order: PP (104.9°) > PET (91.3°) > Nylon (73.1°). The contact angles for the diiodomethane droplets increased in the following order: PP (56.2°) > PET (38.8°) > Nylon (31.6°), as shown in Fig. 1. The surface energy was calculated using the Owens-Wendt-Rabel-Kaelble (OWRK) method^35^, with which the polarity/non-polarity of the surface energy could be calculated. The calculated surface energies were 30.79, 40.91, and 48.68 mN/m for the PP, PET, and Nylon, respectively. High and low surface energies correspond to high and low molecular adsorption powers, respectively. The PP, which has a methyl group on its backbone, exhibited non-polarity properties. The PET, which incorporates an ester group, was found to be of low polarity. Nylon, which incorporates an amide group, proved to have a very high polarity. Thus, in the present study, the conductivity properties of the CB/polymer composites produced by melt-spinning, as well as their influence on the percolation threshold, were studied using polymer matrices of different polarities.

The FT-IR were measured to analyze the chemical structure of the CB and polymer matrix composites. (Fig. S1) Since the CB and the polymer matrix are physically bonded, a large difference could not be appeared by FT-IR patterns.

**Figure 2:**
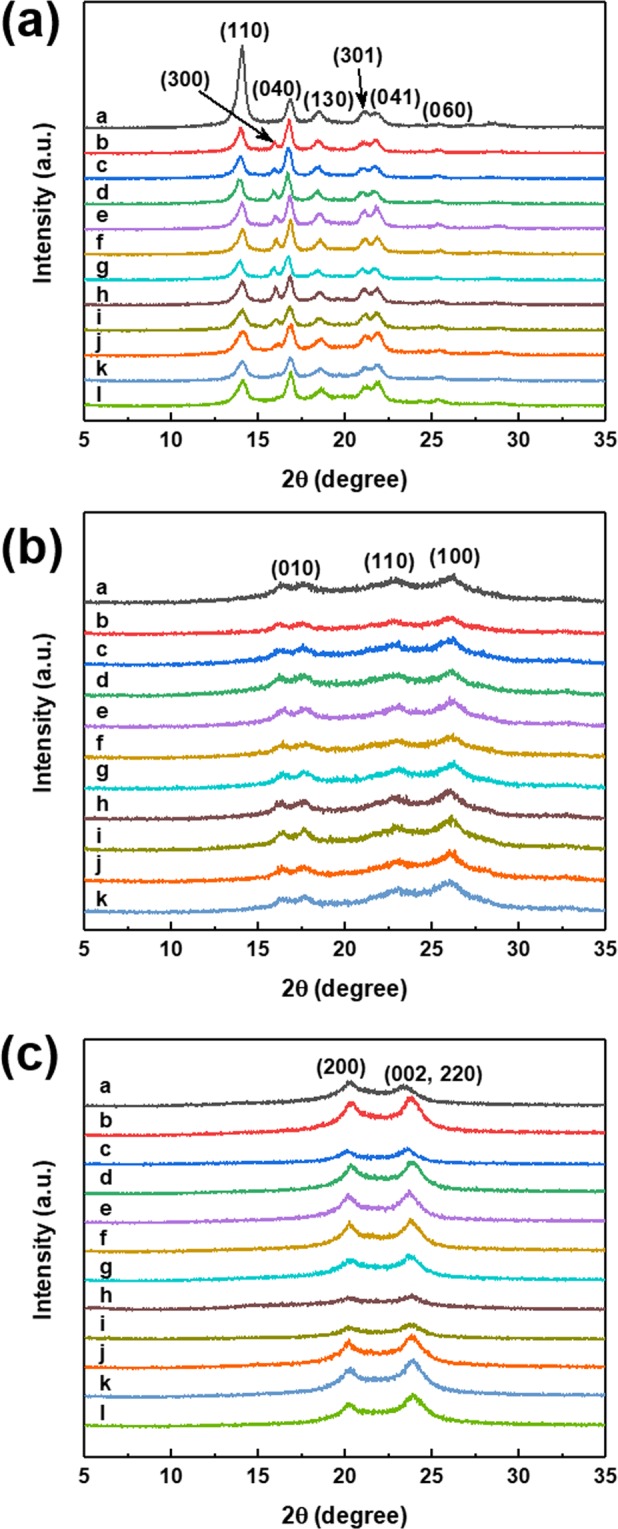
X-ray diffraction patterns. XRD spectra of (**a**) CB/PP, (**b**) CB/PET and (**c**) CB/Nylon composites. (a: neat polymer, b-l is amount of CB content; b: 0.1 wt%, c: 0.5 wt%, d: 0.7 wt%, e:1.0 wt%, f: 3.0 wt%, g:5.0 wt%, h: 7.0 wt%, i: 10.0 wt%, j; 12.0 wt%, k; 15.0 wt%, l: 18.0 wt%).

X-ray diffraction spectra of PP, PET, and Nylon polymer matrix-based carbon black composites are shown in Fig. 2. Figure 2 shows XRD patterns of CB/PP composites. The neat PP is clearly that it shows diffraction peak at the 14.1°, 16.8°, 18.5°, 21.9°, and 25.9° with corresponding of (110), (040), (130), (041), and (060) *α*-form crystal plane and 21.1° related with (301) *β*-form crystal plane. After adding CB filler into PP polymer matrix, the (300) *β*-form crystal plane is newly appeared at 15.9°, which appears to be due to the interaction between PP and CB. This result indicated that the CB incorporation into PP matrix improves crystalline quality by offering more nucleation sites for crystallization. The neat PET (Fig. 2b) shows broad peaks at 17.6°, 22.9°, and 26.3° corresponding of (010), (110), and (100) crystal plane and neat Nylon (Fig. 2c) show two peaks at 20.2° and 23.4° with (200), (002, 220) crystal plane. But, the XRD spectra of CB/PET composite and CB/Nylon composite showed no significant change even after carbon black was added to the neat polymer matrix.

**Figure 3:**
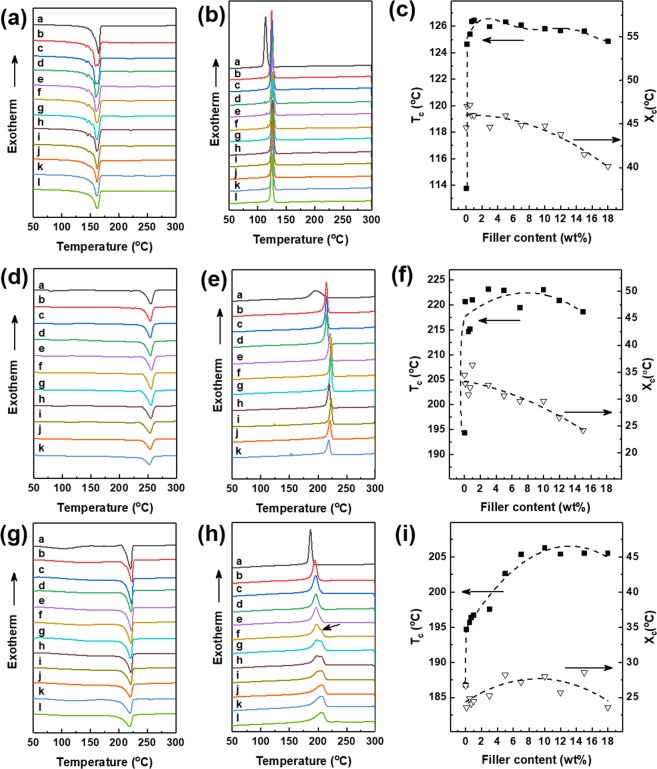
DSC cooling and first heating curves of various materials. (**a,d,g**) Melting thermograms and (**b,e,h**) crystallization thermograms of (**a,b**)CB/PP, (**d,e**) CB/PET, (**g,h**) CB/Nylon composites at a rate of 10 °C/min. And **v**ariation in crystallization temperature (T_*c*_) and change in crystalline content (X_*c*_) of (c) CB/PP, (**f**) CB/PET and (**e**) CB/Nylon composites. (a: neat polymer, b-l is amount of CB content; b: 0.1 wt%, c: 0.5 wt%, d: 0.7 wt%, e:1.0 wt%, f: 3.0 wt%, g:5.0 wt%, h: 7.0 wt%, i: 10.0 wt%, j; 12.0 wt%, k; 15.0 wt%, l: 18.0 wt%).

The cooling curve and heating curve of DSC are shown in Fig. 3 and Table 1. The DSC curves shows that the melting temperature (T_m_) of CB/PP composites (Fig. 3a) are significantly unchanged, while the crystallization temperature (T_c_) of the composite material containing CB as a conductive filler was increased about 10 °C higher than that of neat PP (Fig. 3b). It means that the CB clearly acted as a nucleating agent for the crystallization of PP. The cooling curve and heating curves of CB/PET and CB/Nylon composites showed similar tendency to CB/PP composites. Especially, the CB/Nylon_5 showed a slight broad shoulder peak at ~202 °C and these two peaks merged at CB/Nylon_12. It has been attributed to morphological changes in the crystallite or the melting of small and less stable crystalline units^36,37^.

**Table 1:** Electrical conductivity (S/cm) of CB/polymer composites.

Sample	Carbon black content (wt%)											
	0	0.1	0.5	0.7	1	3	5	7	10	12	15	18
CB/PP	6.9 × 10^–10^	1.6 × 10^−9^	4.1 × 10^−9^	2.2 × 10^−9^	3.0 × 10^−9^	6.3 × 10^–4^	5.8 × 10^–4^	1.9 × 10^−2^	1.9 × 10^−2^	3.2 × 10^−2^	5.1 × 10^−2^	4.1 × 10^−2^
CB/PET	1.6 × 10^−9^	3.6 × 10^−9^	2.0 × 10^−9^	3.4 × 10^−9^	6.6 × 10^−6^	4.9 × 10^–4^	2.4 × 10^−3^	9.4 × 10^−3^	5.2 × 10^−2^	4.3 × 10^−2^	9.5 × 10^−2^	—
CB/Nylon	2.2 × 10^−9^	2.2 × 10^−9^	3.4 × 10^−9^	3.0 × 10^−9^	2.5 × 10^−9^	2.2 × 10^−9^	9.3 × 10^−6^	8.0 × 10^−6^	3.0 × 10^−3^	2.3 × 10^−2^	6.4 × 10^−3^	1.3 × 10^−2^

CB, carbon black; PP, polypropylene; PET, polyethylene terephthalate.

The X*c* refers to crystallinity of PET/polymer composites is shown in Fig. 3c,f and i and Table 2, which were calculated as shows in equation (1)^38^:

1 $${\rm{X}}c=\frac{{\rm{\Delta }}{\rm{H}}c}{{\rm{\Delta }}{\rm{H}}c^\circ \times (1-{\varphi })}\times 100 \% $$

where ΔH*c* is the crystallization enthalpy, ΔH*c*° is the enthalpy of 100% crystalline PP, PET, or nylon, considered as 209.0 J/g^39^, 135.8 J/g^40^ and 230.0 J/g^38^, respectively. And ∅ is the weight ratio of CB.

**Table 2:** Thermal transition parameters of various materials measured by DSC.

Samples	T_*c *_(°C)	T_*m* _(°C)	△H_*c*_ (J/g)	X_*c*_ (%)
PP	113.76	164.69	93.23	44.61
CB/PP_0.1	124.66	163.94	98.26	47.06
CB/PP_0.5	125.39	159.51	98.15	47.20
CB/PP_0.7	126.37	159.94	95.49	46.01
CB/PP_1	126.45	160.32	95.14	45.98
CB/PP_3	125.97	161.47	90.54	44.66
CB/PP_5	126.33	161.06	91.29	45.98
CB/PP_7	126.09	161.70	87.19	44.86
CB/PP_10	125.80	161.98	84.29	44.81
CB/PP_12	125.67	161.75	80.56	43.80
CB/PP_15	125.62	161.42	73.68	41.47
CB/PP_18	124.85	162.21	68.78	40.13
PET	194.36	254.90	48.20	35.49
CB/PET_0.1	220.64	255.52	45.90	33.83
CB/PET_0.5	214.70	254.42	42.83	31.70
CB/PET_0.7	215.17	254.57	44.59	33.07
CB/PET_1	221.02	256.71	50.31	37.42
CB/PET_3	223.14	255.82	44.28	33.62
CB/PET_5	222.89	255.39	40.70	31.55
CB/PET_7	219.43	255.57	38.57	30.54
CB/PET_10	222.97	254.09	37.29	30.51
CB/PET_12	220.86	254.61	32.69	27.35
CB/PET_15	218.60	253.38	28.70	24.86
Nylon	186.75	220.83	61.30	26.65
CB/Nylon_0.1	194.69	222.82	54.13	23.56
CB/Nylon_0.5	195.71	222.36	57.11	24.96
CB/Nylon_0.7	196.39	221.73	55.39	24.25
CB/NylonP_1	196.70	222.34	56.21	24.69
CB/Nylon_3	197.59	221.67	579.8	25.99
CB/Nylon_5	197.57/202.63	221.08	648.4	29.68
CB/Nylon_7	197.69/205.38	221.46	624.6	29.20
CB/Nylon_10	206.33	221.24	643.0	31.06
CB/Nylon_12	205.41	220.88	591.0	29.20
CB/Nylon_15	205.55	220.31	656.0	33.55
CB/Nylon_18	205.57	219.84	542.2	28.75

The crystallization temperature of CB/PP and CB/PET composites increased sharply at 0.1–1 wt% of CB filler and reached a critical value, while CB/Nylon composite gradually increased to adding 3 wt% of CB filter to Nylon. In addition, the X_*c*_ of CB/PP and CB/PET shows a gradually decreased when CB content exceeds 0.5 wt% and 1 wt% respectively, whereas X_*c*_ of CB/Nylon composite does not changed significantly even when the content of CB increases.

### CB/polymer composite electrical conductivity

**Figure 4:**
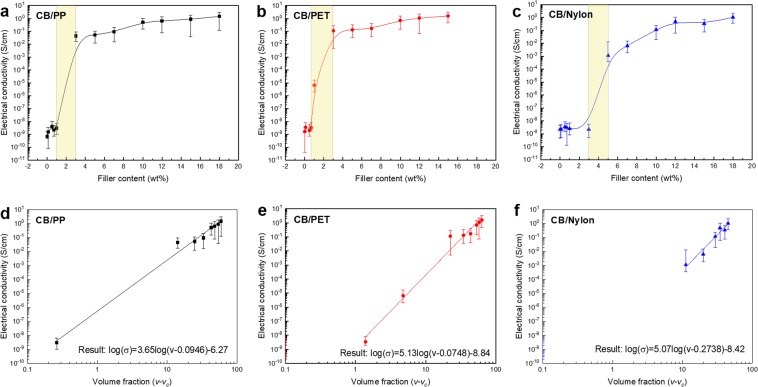
Electrical properties of CB/polymer composites. Electrical conductivity of (**a**) CB/PP, (**b**) CB/PET, and (**c**) CB/Nylon composite as a function of carbon black content. (The percolation threshold appears in the yellow box.) Fitting curve of electrical conductivity versus $(v-{v}_{c})$: (**d**) CB/PP, (**e**) CB/PET, and (**f**) CB/Nylon composite.

The correlation between the electrical conductivity and the properties of polymers has still not been clarified. However, it is thought that the functions of different types of polymers influence the surface energy, and the surface energy influences the electrical conductivity. Figure 4 shows the correlation between the electrical conductivity of each carbon black/polymer composite film and the weight ratio of the additives. The electrical conductivity of CB/Polymer composites was measured 10 times and the mean value was used. Table 2 lists the polymer composites’ electrical conductivities according to each of the conducting additive weight ratios. The change in the composites’ electrical conductivities with the conducting additive weight ratio was such that it increased rapidly at a particular concentration. This concentration is that at which the filler forms interconnections within the matrix. Figure 4(a–c) shows the conductivity of the formed CB/polymer composite film at each concentration. Using each point, a trend line was inserted. The electrical conductivity of the CB/polymer composite increased gradually at low concentrations, and it was confirmed that a percolation threshold arose at a particular concentration, beyond which the electrical conductivity increased rapidly.

The electrical conductivity of the CB/polymer composite was a result of two different mechanisms^41^. The first was a non-ohmic condition generated by the barrier-tunneling effect that occurred in the space between the dispersed carbon black additive in the polymer layers. The second was an ohmic condition, whereby cohesion between the conductive additives starts in the polymer matrix, and a network begins to form such that the current flows more easily (being conducted directly through the linked particles of the conductive additive). The percolation transition occurs as the conducting additive’s concentration approaches the percolation threshold, and the condition transitions from non-ohmic to ohmic. The point of the percolation threshold was different for each CB/polymer composite. The values can be calculated using the power scaling law defined by Equation (2)^42^:

2 $${\rm{\sigma }}=\,{\sigma }_{0}{(v-{v}_{c})}^{t}$$

where σ_0_ is the scaling factor, *v* is the conductive stick volume factor, *v*_*c*_ is the conductive stick volume fraction for the electrical percolation threshold, and *t* is the conductivity exponent.

Figure 4(d–f) shows graphs of electrical conductivity *vs*. $(v-{v}_{c})$. It was confirmed that a percolation threshold appeared for the CB/PP composite at 0.97 wt% (8.46 vol%), for the CB/PET composite at 0.58 wt% (7.48 vol%), and for the CB/Nylon composite at 3.17 wt% (27.38 vol%). That is, it was confirmed that the lowest CB content at which percolation occurred was that in the CB/PET composite, while in the case of the CB/Nylon composite, much larger amounts of CB filler were needed to attain conductivity. As shown in Fig. 4(d–f), a CB/Nylon composite with a low CB volume fraction exhibits no conductivity. Furthermore, the Adj.R-Square value was 0.98685 for CB/PP, 0.97782 for CB/PET, and 0.94811 for CB/Nylon, confirming that the fitting results were very good. After the percolation threshold was attained, all the CB/polymer composites reached the critical point. For a carbon black content of > 12 wt%, regardless of the type of polymer matrix, an electrical conductivity of 1–10^–1^ S/cm arose, as shown in Fig. 4(a–c).

The CB/polymer composite films that used different types of polymer matrix exhibited different electric conductivities due to the different polarities and crystallinities of the polymers. The CB/Nylon composite differed from the CB/PP and CB/PET composites, in that the percolation threshold appeared much later. The amide groups inside the Nylon polymers have a strong polarity and, therefore, cohesion with the carbon black additive occurs, while pores are formed on the surface of the composite. Thus, percolation occurred when the additive content was high. These results are also seen in previous measurements of the contact angle of the polymer and found that the Nylon had the highest surface energy and the strong polarity^43^. Also, the percolation thresholds of CB/polymer composites are similar to those of the crystallization temperatures measured by DSC, and the percolation threshold is also related to the crystallinity.

### CB/polymer composite morphology

An electron microscope examination was undertaken to determine how the morphology of the polymer matrix changed with the carbon black content. The carbon black exhibits a high electron liquidity in a crystallographic hexagonal layer plane, thus making it electrically conductive. The electrical conductivity was found to increase as the particle size of the carbon black became smaller and as it became more highly structured^44^.

**Figure 5:**
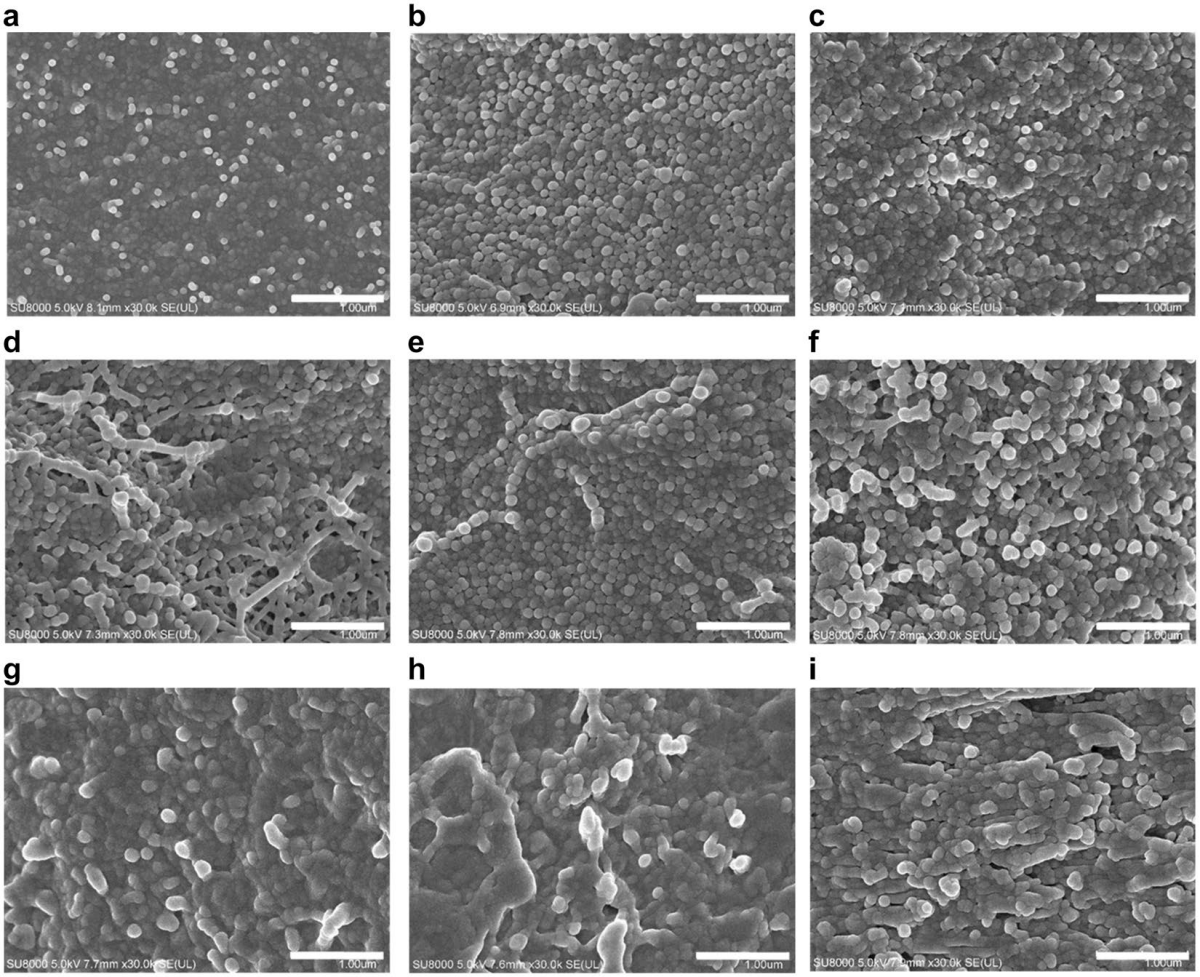
Morphologies of CB/polymer composites. SEM images of CB/PET (**a–c**), CB/PP (**d–f**), and CB/Nylon (**g–i**). The polymer content is 0.1 wt% (**a,d,g**), 3 wt% (**b,e,h**), and 10 wt% (**e,f,i**). Scale bar = 100 μm.

In order to observe the morphology of the CB/polymer composite film, SEM micrograph were captured, as shown in Fig. 5. This SEM imaging was undertaken with two main goals. First, a check was made of how the carbon black was distributed within the polymer resin prior to conductivity being established (0.1 wt%), at the percolation threshold point (3 wt%), and at the critical point (10 wt%). Second, the changes in the morphology of the carbon black and resin for each of the three polymer resins, with different polarities, were observed.

Carbon black/polymer composite films with carbon black percolation thresholds of 0.1 wt%, 3 wt%, and 10 wt% were selected. In the polymer composite with 0.1 wt% carbon black, the connections between the particles of carbon black were incomplete, such that there was no path through which the electrons could flow. For the polymer composite film with a carbon black content of 3 wt%, it was confirmed that a chain structure formed because of the occurrence of inter-cohesion. It can be inferred that the conductivity confirmed earlier was caused by π electron movement in the chain structure. In the polymer composite film with a 10 wt% carbon black content, the carbon black was seen to have coagulated and overlapped. Measurements confirmed that the maximum attainable conductivity was attained once adhesion between the carbon black particles was attained. Beyond this point, increasing the carbon black content further did not affect the conductivity.

Each of the three types of polymer matrices exhibited a different polarity due to their different chemical structures. In the CB/nylon composite with an amide group, such that it exhibited a strong polarity, the nylon polymer generally covered the carbon black. The nylon prevented the particles of carbon blacks from coming into contact with each other, and thus played a major role in preventing the percolation threshold being attained at low carbon black concentrations. Among the CB/polymer composites, the best carbon black dispersion was observed in the CB/PET composite. In fact, the CB/PET composite exhibited the lowest percolation threshold, which was a result of the formation of a contact surface due to the increase in the surface area caused by the even dispersion. The correlations between the CB/polymer composites’ percolation thresholds and morphologies were confirmed through conductivity measurements.

**Figure 6:**
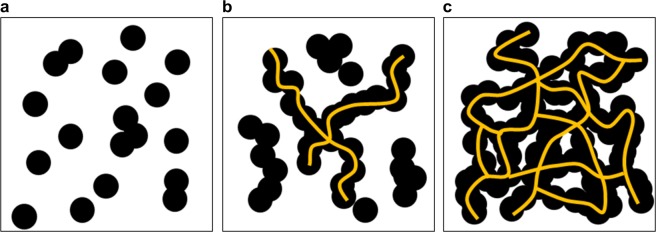
Schematics of percolation thresholds of CB/polymer composites. Orientations of carbon black in polymer matrix: carbon black was (**a**) disconnected, (**b**) partially connected, or (**c**) fully connected.

The CB/polymer composites’ electrical conductivities and morphologies were synthesized and their percolation thresholds were schematized as shown in Fig. 6. The electrical conductivity of the CB/polymer composite changed from the insulating region (Fig. 6(a)) to the conducting region (Fig. 6(c)), based on the charge amount of certain CB particle limits. When this point was exceeded, even if the amount of additive was increased only slightly, the resistance of polymer/additive composite rapidly decreased. Figure 6(b) shows that, if the amount of conductivity additive was further increased, the critical threshold was attained and the resistance fell and stabilized at zero, subsequently remaining at that value. From the perspective of electrical conductivity, when a uniform dispersion occurred within the CB/polymer composite, a conductive path was formed by the carbon black. Even the addition of small amounts of carbon black can cause the electrical conductivity to increase rapidly.

### Conductivity analysis of CB/PET fibres and textiles

**Figure 7:**
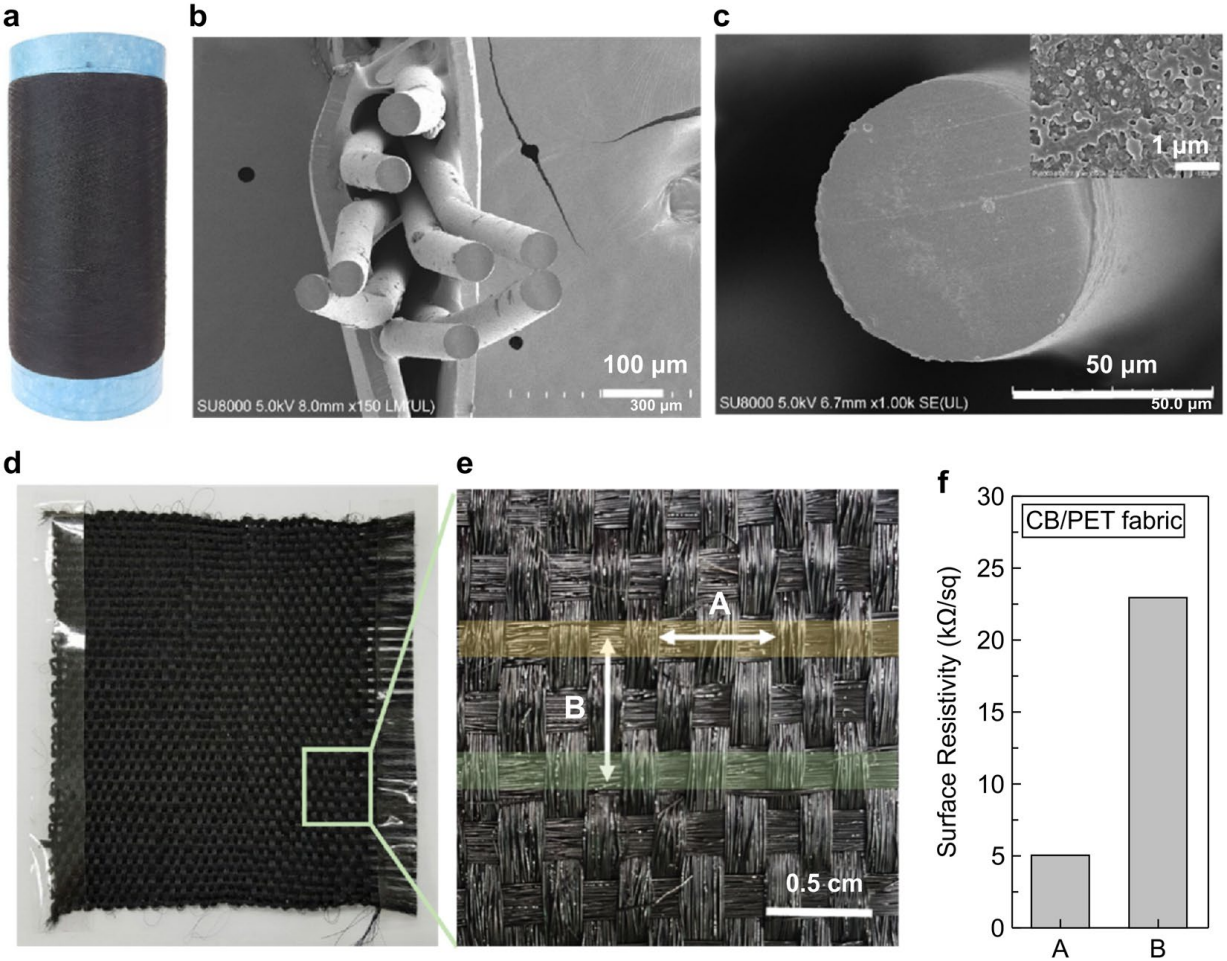
(**a**) Photograph and (**b**) SEM images of CB/PET composite fibres, (**c**) magnified (**b**) (inset: further magnified (**b**)), (**d**) CB/PET composite fabric, (**e**) magnified (**d**), (**f**) surface resistivity of CB/PET composite fabric.

The CB/PET composite with the lowest percolation threshold and best conductivity was selected for application to the manufacture of fibres and textiles. A CB/PET composite fibre with a 3 wt% carbon black content, appropriate for creating a flexible textile with excellent electric conductivity, was manufactured. The CB/PET composite fibre was manufactured using a twin-screw extruder, with the manufacturing process being similar to that used to produce the film, as described above. The composite material, after being forced through the die, was wound onto the winder at high speed (400 m/min). The resulting black conductive fibre is shown in Fig. 7(a). The cross-section of the CB/PET composite fibre was checked using SEM. The diameter was confirmed to be very uniform, at 60–70 μm, as shown in Fig. 7(b). Figure 7(c) shows that the fibre appears smooth and round. High-magnification images of cross-sections of a single fibre and the CB/PET composite film showed that the carbon black was uniformly dispersed throughout the PET polymer matrix. Figure 7(d) shows a plain-weave fabric made from the CB/PET fibre. The magnified image shown in Fig. 7(e,f) shows the intersecting warp and weft of the plain-weave fabric. The CB/PET composite fibre surface resistance in the A direction (30 strands of CB/PET yarn) was 5 kΩ/sq (0.0023 S/cm), and 23 kΩ/sq (0.0007 S/cm) in the B direction (in the CB/PET yarn-to-yarn interstices). The resistance is higher in the B direction because the resistance is measured across the lengths of yarn, rather than along them. The compressed CP/PET composite fibre exhibits a low resistance and excellent electrical conductivity. It is thought that fabrics manufactured using this process could be used in antistatic applications^2^.

## Conclusions

In the present study, CB/polymer composites were successfully produced by adding carbon black to a matrix of PP, PET, or Nylon by melt compounding method. The percolation threshold, an electrical property that depends on the polarity and crystallinity of the polymer, were examined. Through SEM imaging, it was showed that the dispersion of the carbon black was most uniform in the CB/PET composite. The CB filler can act as nucleating agent in polymers, which increased the crystallization temperature of neat PP, PET and Nylon polymer and a newly generated *β*-form crystal plane at PP polymer. Electrical conductivities of CB/polymer composites increased with an increase carbon black loading. The CB/PET composite a high electrical conductivity of 9.5 × 10^−2^S/cm and a low percolation threshold of 0.58 wt% (7.48 vol%). And, in the case of the Nylon-based composite, which had a very high polarity, the inter-cohesion between the polymer and the carbon black was very strong, with hydrogen bonding arising within the polymer resin, preventing the flow of an electric current. However, nonpolar PP and relatively weak polar PET lead to the current flows well and results in a low percolation threshold value. Furthermore, fabrics woven from the CB/PET composite fibres had an excellent electrical conductivity of 5 kΩ/sq (2.3 × 10^−3^ S/cm), indicating that they would be well suited for use as antistatic materials.

## Methods

### Materials

For our experiments, we used polypropylene (PP; HP552R^®^, Polymirae Co., Ltd., Korea), polyethylene terephthalate (PET; JSD 588^®^, Huvis Co., Korea), and Nylon (Nylon 6; 1011 BRT^®^, Hyosung Co., Korea) as the polymer matrix. The specific surface area of the carbon black used as the conductivity additive was 1270 m^2^/g Ketjen Black EC 600JD^®^ (AkzoNobel, Netherlands) to which oil had been added at a rate of 4.8–5.1 ml/g. Prior to extrusion, any moisture was removed by subjecting the materials to vacuum drying at 100 °C for 10 h.

### Processing

PP, PET, and Nylon were each used as the polymer matrix. To create a conductive polymer composite in which carbon black was evenly dispersed, the carbon black and the polymer matrix were placed in a 2 L beaker and carbon black and polymer was mixed several times by hand to concentrations of 0.1, 0.5, 0.7, 1.0, 3.0, 5.0, 7.0, 10.0, 12.0, 15.0, and 18 wt%, Then a twin-screw extruder (BA-11, Bautek Co., Korea) with a diameter of 11 mm, fitted with a 1-mm nozzle with a length to diameter (L/D) ratio of 3.64, was used to prepare the CB/polymer composites. The carbon black/polymer mixture was placed in the extruder’s hopper and used to perform melt-spinning. (In the case of the PET, melt-spinning with a carbon black concentration of 18 wt% proved impossible, so no experiments were performed beyond a concentration of 15 wt%). The sample name is CB/polymer_X and X is the content of CB filler.

**Figure 8:**
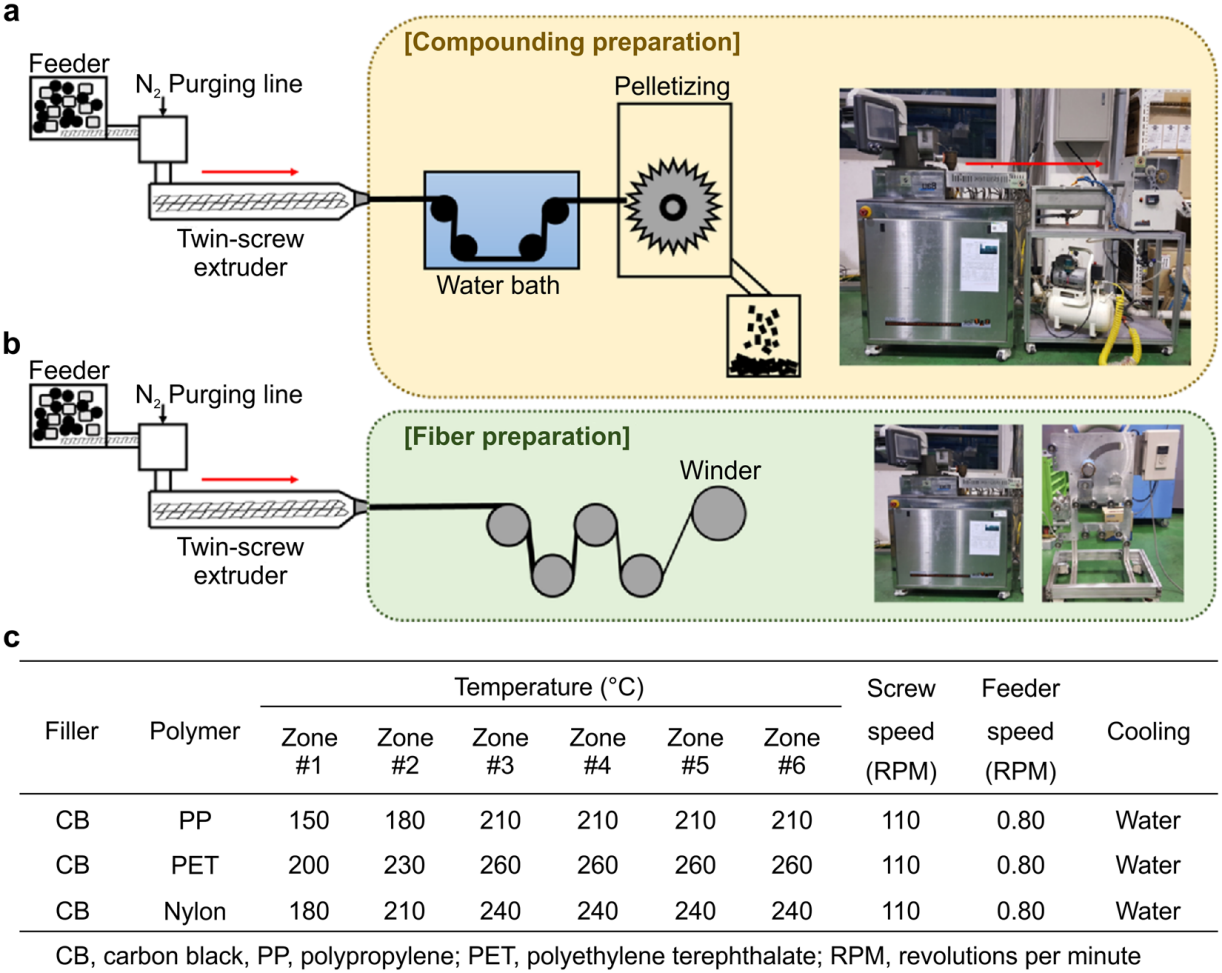
A schematic representation for the compounding and fibre preparation with condition. Schematic and photographs of (**a**) compounding, (**b**) fibre preparation and table of (**c**) compounding condition.

The CB/polymer composite film was fabricated using a twin-screw extruder. As listed in Fig. 8, the temperatures of each of the six regions of the extruder were controlled, and the screw and feeder speeds were adjusted appropriately for each composite.

Figure 8(c) lists the temperature of the extruder, the rotational speed of the screw, and the speed of the feeder. Inside the extruder’s cylinder, the composites melt-mixed before passing through the dies to form strands. They were then immediately passed through cold water to solidify them, after which a pelletizer was used to transform the strands into pellets (Fig. 8(a)).

To measure the electrical conductivity of the CB/polymer film produced by melt-spinning, a hot-plate hydraulic press (4122^®^, Carver, Inc., Indiana, USA) was used. A 5×5 cm film was fabricated by adding heat and pressure for 5 min at 40 MPa, with the CB/PP composite at 220 °C, the CB/PET composite at 270 °C, and the CB/Nylon composite at 250 °C.

The CB/PET composite fibre was fabricated by using a twin-screw extruder with a single 3-mm die. The maximum ratio of carbon black (3 wt%) that could be incorporated while still producing a conductive, thin, and flexible fibre was added to a PET matrix (Fig. 8(b)). The spinning conditions (temperature, screw speed, and feeder speed) were the same as those used when creating the CB/PET compound. The resulting CB/PET composite was immediately wound onto the winders at a speed of 400 m/min (Fig. 8(b)).

**Figure 9:**
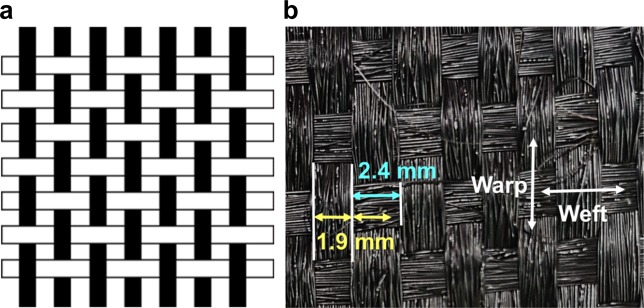
(**a**) Schematic of plain weave using CB/PET fibres and (**b**) properties of woven fabric. (■: warp, □: weft).

The CB/PET composite fibre was used to manufacture a fabric, which featured a plain weave with an alternating warp and weft, as shown in Fig. 9(a). An untwisted CB/PET composite yarn was manufactured using 30 fibre strands, each with a diameter of 60–70 µm for both the warp and weft. The density of the CB/PET fibre was 9×9 fibres/inch. The width of the yarn was 1.9 mm, and the distance between each thread was 2.4 mm, as shown in Fig. 9(b).

### Characterizations

The degree of dispersion of the carbon black in the CB/polymer composite film and fibre polymer matrix was observed using field emission scanning microscopy (FE-SEM; SU8000, Hitachi Ltd., Tokyo, Japan). Samples were cut in liquid nitrogen, after which a cross-section was checked. The contact angle of the polymer film was measured at room temperature using a KRÜSS DSA 100 S^®^ (KRÜSS, Hamburg, Germany). Water and diiodomethane were used. Using 4.5 μl of water and a 1.5 μl drop of diiodomethane, the contact angle between the polymer surface and the solution was measured. The surface energy was calculated using DSA3 software which runs on the DSA100S^®^. Thermal properties including melting point and crystallizaiton temperature were measured under nitrogen flow using a different scanning calorimeter (DSC) instrument (TA Instrument Q100 DSC, TA Instrument, NewCastle, USA), it was heated from 50 °C to 300 °C with a heating rate of 10 °C/min. The X-ray diffraction powder pattern (XRD, Rigaku, Japan) was measured using a D/MAX UltimaIII diffractometer with thetha-theta goniometer equippped with Cu K_α_ radiation (λ = 1.54056 Å). The scan rate is 2°/min between scanning angle 2θ = 10–80°. Fourier transform infrared (FTIR) spectra were recorded on Nicolet Nexux 760 (Thermo Fisher Scientific, USA).

The conductivities of all the CB/polymer composite films were found to be lower than 10^−5^ Ω/cm, as measured using a Keithley 6517B^®^ 2-point probe high-resistance meter (Keithley Instruments, Inc., Cleveland, OH, USA) with an 8009 resistivity test fixture. All the measurements were performed according to the ASTM-D-257 standard resistance measuring method. Prior to the measurements being taken, 100 V was applied for 1 min to allow the current to stabilize. When the electrical conductivities of CB/polymer composite film and the fabrics were higher than 10^−5^ Ω/cm, the four-point probe method (CMT-100S^®^, AIT Co., Ltd., Suwon-city, Korea) was used to measure the electrical conductivity. The diameter of each of the probe’s pins was 0.25 mm, and the distance between the pins was 3 mm. However, the electrical conductivity of the CB/polymer composite fibre itself was measured using the two-point probe method.

With the two-point probe method, the conductivity is calculated using the following equations:

3 $${\rm{\sigma }}(\frac{{\rm{S}}}{{\rm{cm}}})=\frac{1}{{\rho }_{sheet}(\frac{ohm}{sq})\cdot d(cm)}$$

4 $${\rho }_{sheet}(\frac{ohm}{sq})=\frac{V}{I}\cdot C.F.$$

where ${\rm{\sigma }}$ is the electrical conductivity as measured using either the 2- or 4-point probe method, *d* is the thickness of the sample, V is the voltage, I is the measured current, and C.F. is the calibrating constant. The value of C.F. is influenced by the sample size, thickness, and temperature. For the present study, C.F was set to 53.4 and 4.532 for the 2- and 4-point probe methods, respectively^34,45^.

## Supplementary information

Electronic supporting information

## Data Availability

All data generated or analyzed during this study are included in this published article.
