# Supplementary material for: Electrical percolation threshold of carbon black in a polymer matrix and its application to antistatic fibre
Source: Sci Rep. 2019 Apr 19;9:6338. doi: 10.1038/s41598-019-42495-1 (PMC6474880; doi:10.1038/s41598-019-42495-1)
Supplement: Supplementary file 1 — Electronic supporting information [file 41598_2019_42495_MOESM1_ESM.docx]

**Electronic Supporting Information (ESI) for:**

**Electrical percolation threshold of carbon black in a polymer matrix and its application to antistatic fibre**

Hyun-Jung Choi^1^, Moo Sung Kim^1^, Damiro Ahn^1^, Sang Young Yeo^1,*^, So Hee Lee^2^

^1^Technical Textile and Materials R&D Group, Korea Institute of Industrial Technology, 143 Hanggaulro, Sangnok-gu, Ansan-si, Gyeonggi- do, 15588, Republic of Korea

^2^Department of Clothing and Textiles, Gyeongsang National University, 501 Jinju-daero, Jinju-si, South Gyeongsang Province, 52828, Republic of Korea

***Corresponding authors**

Sang Young Yeo, Technical Textile and Materials R&D Group, Korea Institute of Industrial Technology, Gyeonggi-do, Korea.

Phone number: +82-31-8040-6068; Fax number: +82-31-8040-6080. E-mail: miracle@kitech.re.kr


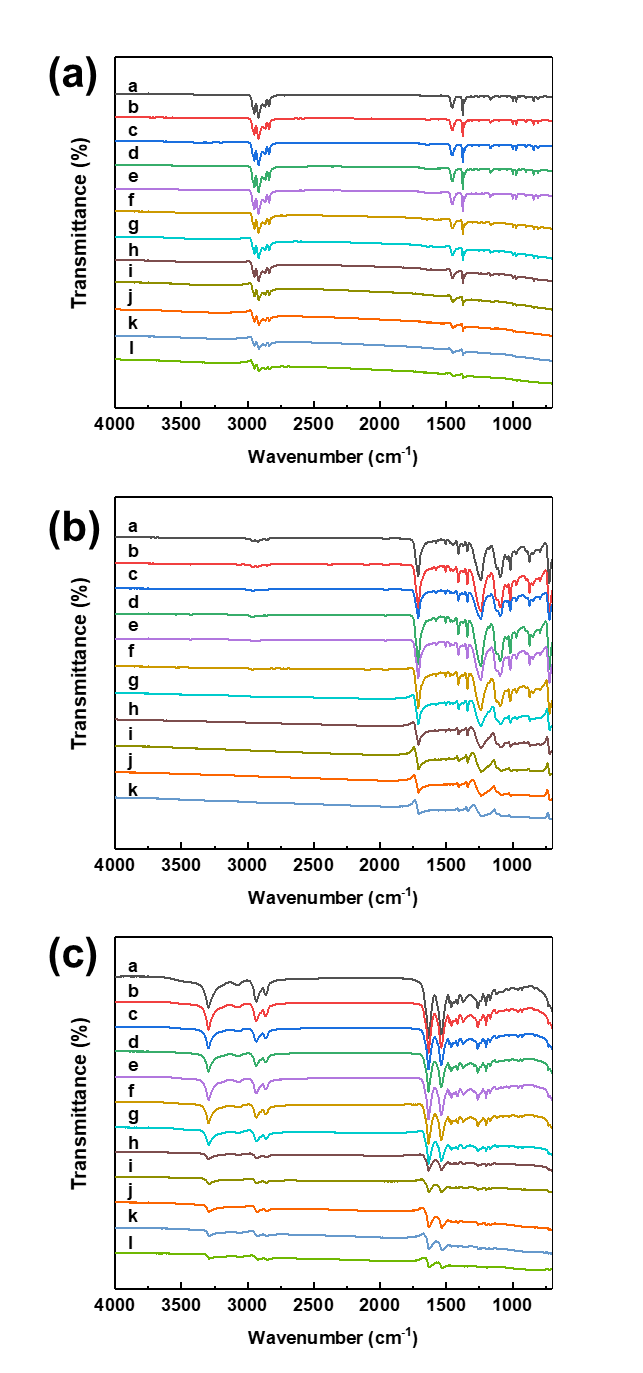


**Fig. S1.** FT-IR spectra of (a) CB/PP, (b) CB/PET and (c) CB/Nylon composites. (a: neat polymer, b-l is amount of CB content; b: 0.1wt% , c: 0.5wt%, d: 0.7wt%, e:1.0 wt%, f: 3.0 wt%, g:5.0 wt%, h: 7.0 wt%, i: 10.0 wt%, j; 12.0 wt%, k; 15.0 wt%, l: 18.0 wt%)
